# Low doses of widely consumed cannabinoids (cannabidiol and cannabidivarin) cause DNA damage and chromosomal aberrations in human-derived cells

**DOI:** 10.1007/s00204-018-2322-9

**Published:** 2018-10-19

**Authors:** Chiara Russo, Franziska Ferk, Miroslav Mišík, Nathalie Ropek, Armen Nersesyan, Doris Mejri, Klaus Holzmann, Margherita Lavorgna, Marina Isidori, Siegfried Knasmüller

**Affiliations:** 1 Dipartimento di Scienze e Tecnologie Ambientali, Biologiche e Farmaceutiche, Università della Campania, L. Vanvitelli, Via Vivaldi 43, 81100 Caserta, Italy; 2 Department of Internal Medicine 1, Institute of Cancer Research, Medical University of Vienna, Borschkegasse 8A, 1090 Vienna, Austria

**Keywords:** CBD, CBDV, Genotoxicity, SCGE assay, MN assay

## Abstract

Cannabidiol (CBD) and cannabidivarin (CBDV) are natural cannabinoids which are consumed in increasing amounts worldwide in cannabis extracts, as they prevent epilepsy, anxiety, and seizures. It was claimed that they may be useful in cancer therapy and have anti-inflammatory properties. Adverse long-term effects of these drugs (induction of cancer and infertility) which are related to damage of the genetic material have not been investigated. Therefore, we studied their DNA-damaging properties in human-derived cell lines under conditions which reflect the exposure of consumers. Both compounds induced DNA damage in single cell gel electrophoresis (SCGE) experiments in a human liver cell line (HepG2) and in buccal-derived cells (TR146) at low levels (≥ 0.2 µM). Results of micronucleus (MN) cytome assays showed that the damage leads to formation of MNi which reflect chromosomal aberrations and leads to nuclear buds and bridges which are a consequence of gene amplifications and dicentric chromosomes. Additional experiments indicate that these effects are caused by oxidative base damage and that liver enzymes (S9) increase the genotoxic activity of both compounds. Our findings show that low concentrations of CBD and CBDV cause damage of the genetic material in human-derived cells. Furthermore, earlier studies showed that they cause chromosomal aberrations and MN in bone marrow of mice. Fixation of damage of the DNA in the form of chromosomal damage is generally considered to be essential in the multistep process of malignancy, therefore the currently available data are indicative for potential carcinogenic properties of the cannabinoids.

**Electronic supplementary material:**

The online version of this article (10.1007/s00204-018-2322-9) contains supplementary material, which is available to authorized users.

## Introduction

**Fig. 1:**
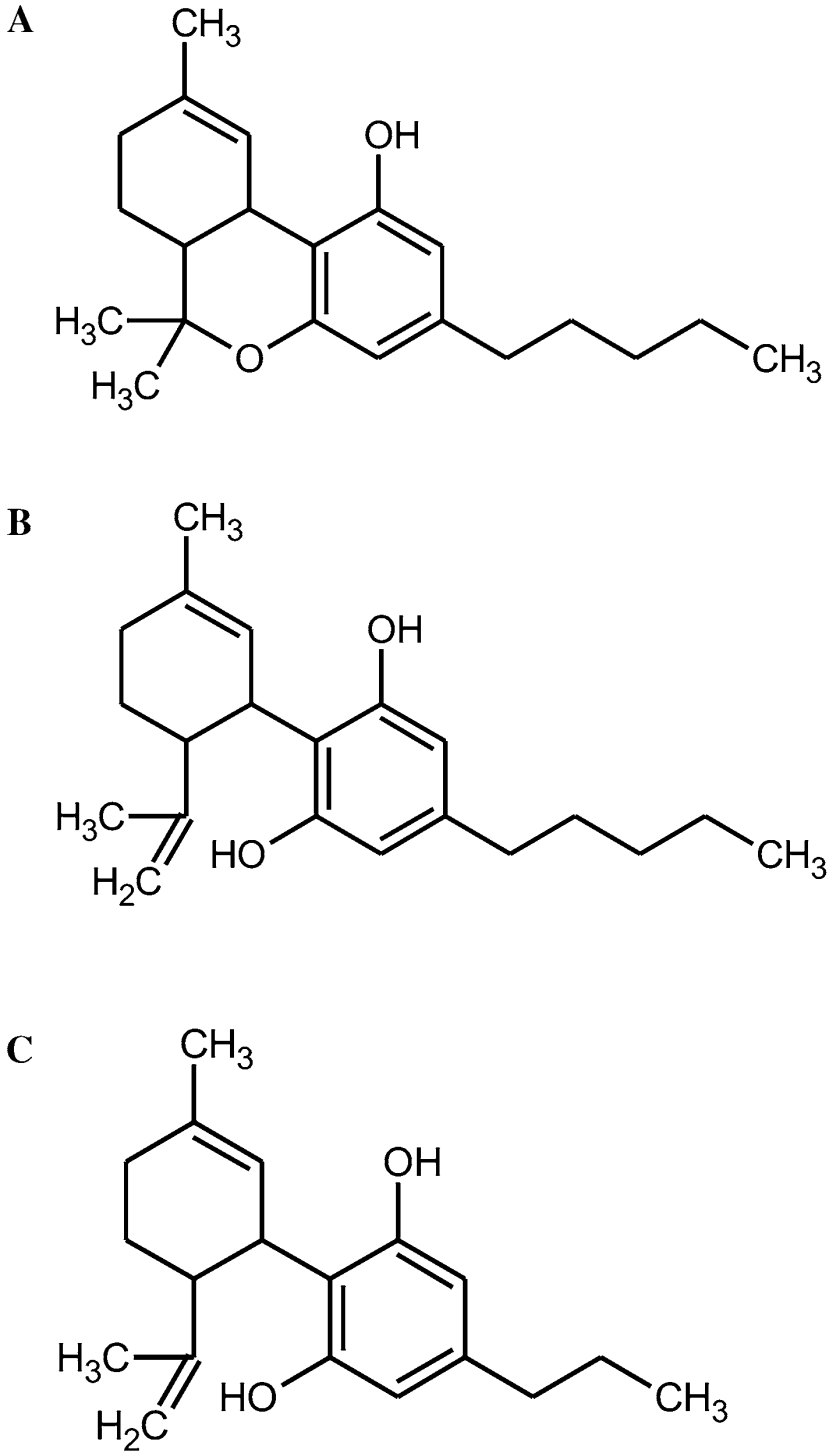
Chemical structure of the test compounds. **a** ∆9-THC (CAS Nr. 1972-08-3), **b** CBD (CAS Nr. 13956-29-1), **c** CBDV (CAS Nr. 24274-48-4) is a propyl derivative of CBD

Cannabidiol (CBD) and cannabidivarin (CBDV) are naturally occurring cannabinoids which are widely consumed. CBD is structurally related to ∆9-tetrahydrocannabinol (THC) and occurs together with its propyl analogue (CBDV) in *Cannabis sativa* and *C. indica* plants. Both agents cause a variety of pharmacological effects but do not have the psychotropic properties which are characteristic for THC. CBD and CBDV are antiepileptic, anticonvulsant, and antipsychotic (Fernández-Ruiz et al. 2013; Hill et al. 2012; Rosenberg et al. 2015; Ujvary and Hanus 2016); furthermore, it was postulated that the former compound prevents inflammation (Borrelli et al. 2009) and may act as an anti-carcinogen (Aviello et al. 2012; Massi et al. 2013). Figure 1a–c depict the structure of the compounds.

It was repeatedly stressed that the use of CBD is safe and that it is well-tolerated by humans (Bergamaschi et al. 2011; Iffland and Grotenhermen 2017). At present, a large number of extracts and oils of cannabis plants which contain CBD and CBDV and low levels of THC are marketed in European countries and also in the US, and several clinical trials concerning their health effects are in progress (Fasinu et al. 2016). The preparations are mainly sold via the internet (64%) and in hemp shops (17%), but also in drugstores and pharmacies (Borchardt 2018). The sales of these products are booming at present. According to Forbes Magazine, the market increased by 700% in recent years (http://www.forbes.com) and it is stated in a report of the market intelligence of the Hemp Business Journal that sales will exceed 2.1 Billion USD in 2020 (NSE 2018).

Since CBD and CBDV are natural substances, the current legislation does not foresee toxicological testing which is obligatory for pharmaceutical drugs and no potential long-term effects such as induction of cancer, infertility, and malformations in the offspring have been investigated. These latter effects may be due to damage to the genetic material, but only few studies which date back to the 1980s were realized. Zimmerman and Raj (1980) tested CBD in mice and found evidence for induction of micronuclei (MNi) in bone marrow cells of mice, which are formed as a consequence of structural and numerical chromosomal aberrations in bone marrow cells. Furthermore, the same authors reported increased rates of chromosomal aberrations (CA) in the same target tissue by CBD (Zimmerman and Raj 1980).

The aim of the present study was to investigate if CBD and CBDV cause damage to the genetic material in human-derived cells, under conditions which reflect the situation in users. We investigated the effects of these compounds in single cell gel electrophoresis (SCGE) assays which are based on the measurement of DNA migration in an electric field and reflect single and double strand breaks, as well as apurinic sites (Azqueta and Collins 2013). The SCGE technique is among the most widely used methods in genetic toxicology (Neri et al. 2015). The compounds were tested in a human-derived hepatoma cell line (HepG2) which reflects the metabolism of xenobiotics better than other cell lines currently used (Knasmuller et al. 1998). Since CBD and CBDV preparations are mainly consumed orally, additional experiments were conducted with TR146 cells which are derived from the buccal epithelium (Rupniak et al. 1985). To elucidate if (repairable) DNA damage (which is detected in the SCGE experiments) leads to formation of persisting chromosomal mutations, MN cytome experiments were performed, to monitor induction of MNi, which reflect structural and numerical chromosomal aberrations and other nuclear anomalies (Nbuds and NPBs), which are formed as a consequence of gene amplifications and dicentric chromosomes (Fenech 2007).

To characterize the molecular mechanisms, by which the compounds cause genetic instability, additional experiments were performed which enable the assessment of formation of oxidized purines and pyrimidines by use of a modified protocol of the SCGE assay with lesion-specific enzymes according to the protocol of Collins and Dušinská (2002). Finally, a series of experiments with liver homogenate (S9 mix) was conducted to find out if drug-metabolizing enzymes are involved in the activation of the compounds.

## Materials and methods

### Chemicals

Low melting point agarose (LMPA) and normal melting point agarose (NMPA) were obtained from Gibco (Paisley, UK). Inorganic salts, dimethyl sulfoxide (DMSO), methanol, propidium iodide, hydrogen peroxide, triton X-100, trizma base, bovine serum albumine (BSA), cyclophosphamide, cytochalasin B, Dulbecco’s phosphate-buffered saline (DPBS), fetal calf serum (FCS), trypsin–EDTA, Na_2_-EDTA, 4-(2-hydroxyethyl)-1-piperazine-ethanesulfonic acid (HEPES), trypan blue and cyclophosphamide (CP) were purchased from Sigma-Aldrich (Steinheim, Germany).

### Test compounds

Cannabidiol (CBD, CAS 13956-29-1, purity 99.95%) was obtained from LGC Standards GmbH (Germany) and cannabidivarin (CBDV, CAS 24274-48-4, purity 99.80%) from Sigma-Aldrich (Milan, Italy). Both compounds were dissolved in methanol.

### Cultivation of cell lines (HepG2 and TR146)

The human hepatoma cell line (HepG2) was provided by F. Darroudi (Department of Toxicogenetics, Leiden University Medical Centre, the Netherlands). The cells were grown in Eagle’s Minimal Essential Medium (EMEM, Sigma-Aldrich, Steinheim, Germany) supplemented with 1.0 mM sodium pyruvate (MNP medium) and 10% FCS. The fifth to eighth passages from stock cultures (in liquid nitrogen) were used for the SCGE and MN experiments.

The human cell line TR146 which is derived from buccal epithelial tissue (Rupniak et al. 1985) was obtained from J. G. Rheinwald (Dermatology Institute of Boston, MA USA). The cells were cultivated in Dulbecco’s modified Eagle Medium (DMEM, Sigma-Aldrich, Steinheim, Germany) with 10% FCS. The cells were stored in liquid nitrogen. The fourth to the sixth passage were used for the genotoxicity experiments.

Both cell lines were cultivated under standard conditions (37 °C, humidified atmosphere, 5% CO_2_). The media were changed every 2–3 days. When the cultures had reached confluency, the cells were washed with DPBS, detached with trypsin/EDTA, centrifuged and sub-cultured.

### Measurements of cytotoxic effects

The viability of the cells was determined with a CASY^®^ cell counter (Schärfe-System GmbH, Reutlingen, Germany). This method is based on the determination of electric potential differences (Lindl et al. 2005). Briefly, cells (2.0 × 10^5^ cells/well) were seeded in 24-wells plates (Becton, Dickinson and Company, NJ, USA) in media which contained different concentrations of CBD (0.22–162 µM) and CBDV (0.66–162 µM) for 3 h or 24 h. In all experiments, solvent controls and positive controls were included. The cells were detached with trypsin–EDTA, centrifuged (200*g*, 5 min, 21 °C) and suspended in 1.0 mL medium. 50.0 µL of these suspensions were transferred to CASY-cups (OLS OMNI Life Science GmbH & Co. KG, Bremen, Germany). For each experimental point, two independent experiments were performed and means ± standard deviations were calculated. Additionally, we tested the viability of the cells after exposure to the test compounds with the trypan blue exclusion technique (Lindl and Bauer 1994).

### Single cell gel electrophoresis (SCGE) assays (standard conditions)

The experiments were conducted according to the protocol of Tice et al. (2000) under alkaline conditions. Only cultures with a viability ≥ 80% were evaluated in SCGE assays.

The indicator cells (2.0 × 10^5^ cells/well) were transferred into 24-well plates which contained 1.0 mL medium with different concentrations of CBD and CBDV. The cells (HepG2) were exposed to the test compounds for 3 h and 24 h (3 h: dose range 0.66–54, 24 h: dose range 0.22–18 µM). TR146 cells were treated with the cannabinoids for 3 h (dose range 2.00–54 µM). In all experiments, solvent controls (methanol) and positive controls (H_2_O_2_, 50 µM) were included. The pellets were resuspended in low melting point agarose (0.5% LMPA). Subsequently, the cells were spread on pre-coated agarose slides (1.5% NMPA) and lysed in the dark at 4 °C for at least 60 min. After 30 min of unwinding under alkaline conditions (pH > 13), electrophoresis was carried out for 30 min (300 mA, 1.0 V/cm, at 4 °C); neutralization was performed twice for 8 min. Air-dried slides were stained with propidium iodide (10 µg/mL). Subsequently, the percentage of DNA in the tails was measured by use of an image analysis system (Comet IV, Perceptive Instruments Ltd., Burry St. Edmunds’, UK). For each experimental point, two slides were prepared and 50 nuclei were evaluated randomly on each slide. Two independent experiments were performed.

In experiments with rat liver homogenate (S9), 10 µL S9 mix was added to the inoculation mix (final protein concentration 30 mg/mL). MUTAZYME™ rat S9 mix (10%) was purchased from TrinovaBiochem GmbH (Giessen, Germany). MUTAZYME™ consists of Aroclor 1254-induced male Sprague Dawley rat liver S9 which was lyophilized with NADP, d-glucose-6-phosphate, MgCl_2_/KCl in pH 7.4 sodium phosphate buffer. The mixtures were incubated for 3 h (37 °C; shaking 250 rpm). Subsequently, the cells were washed and processed as described above. Two independent experiments were performed. For each experimental point, two slides were prepared and 50 nuclei were evaluated randomly from each slide.

### Single cell gel electrophoresis (SCGE) assays with lesion-specific enzymes

The impact of the drugs on the formation of oxidized DNA bases was monitored in additional experiments with lesion-specific enzymes. Formamidopyrimidine DNA glycosylase (FPG) and endonuclease III (ENDO III) were purchased from Sigma-Aldrich (Steinheim, Germany). To define the optimal concentrations of the enzymes, calibration experiments were carried out before the main experiments [for details see Collins et al. (1997), data not shown].

The cells (HepG2) were exposed to the test compounds as described above. The experiments with lesion-specific enzymes were conducted according to the protocol of Collins and Dusinska (2002).

After lysis, the slides were washed for 8 min twice with enzyme reaction buffer (40 mM HEPES, 0.1 M KCl, 0.5 mM Na_2_EDTA, 0.2 mg/mL BSA, pH 8.0). Subsequently, the nuclei were treated either with 50 µL of the enzyme solutions or with the enzyme buffers. The incubation time for experiments with FPG was 30 min and for Endo III 45 min at 37 °C, respectively. After the treatment, electrophoresis was carried out under standard conditions (30 min, 300 mA, 1.0 V/cm, at 4 °C, pH > 13). After electrophoresis, the slides were processed and evaluated as described above. Two independent experiments were performed. For each experimental point, two cultures were set up. From each culture, two slides were prepared and 50 cells were evaluated from each slide.

### Cytokinesis-block micronucleus (CBMN) assays with HepG2

The experiments were conducted as described by Koller et al. (2014). Briefly, 5.0 × 10^5^ cells/well were seeded in 6-well plates with 3.0 mL medium and allowed to attach overnight. Subsequently, the medium was removed after washing with DPBS. The cells were treated with different concentrations (0.07–2 µM) of the test compounds in serum-free medium for 3 h. Cyclophosphamide (final concentration 500 µg/mL) was used as a positive control. After treatment of the cells with the drugs for 3 h, they were washed with PBS. Subsequently, they were incubated with cytochalasin B (3.0 µg/mL) to block cytokinesis and DMEM (with 10% FCS) for 27–28 h. Then, the cells were washed, trypsinized and harvested. Slides were prepared with the cyto-centrifugation method (Fenech 2007). After drying, they were stained with Diff Quick (Dade Behring, Deerfield, IL, USA) and fixed with Entellan (Sigma-Aldrich, Steinheim, Germany).

Per experimental point, two cultures were made. Four slides were prepared and 2000 cells were evaluated. Different endpoints were scored namely, mono-nucleated, binucleated (BN) and multi-nucleated cells as well as the rates of binucleated cells with MN (BN–MN), the total number of MN in binucleated cells (MNi), nuclear buds (Nbuds), and nucleoplasmatic bridges (NPBs). The cytokinesis-block proliferation indices (CBPI) were calculated with 500 cells according to the formula CBPI = [*M*1 + 2*M*2 + 3(*M*3 + *M*4)]/*N* (*N* is the total number of scored cells), *M*1–*M*4 refers to the number of cells with one to four nuclei (OECD 2016). The toxicity of the compounds was indirectly assessed by the assumption that a CBPI of 1.0 corresponds to 100% cytotoxicity (OECD 2016). Five concentrations of each drug were used to determine the CBPI values. Two independent experiments were performed; per experimental point, four slides were prepared and 2000 cells were evaluated. In agreement with OECD guideline #487 (OECD 2016), only doses causing less than 60% cytotoxicity were analyzed with regard to formation of nuclear anomalies. Early necrotic cells, characterized by pale cytoplasm and presence of many vacuoles, and late necrotic cells, identified by loss of cytoplasm and damaged nuclear membranes, were scored according to the protocol of Fenech (2007). Apoptotic cells were identified morphologically by changes in the chromatin structure and by nuclear fragmentation (Fenech 2007).

### Statistical analyses

All results were analyzed with the GraphPad Prism 5 software system (LaJolla, CA, USA). The data from the SCGE experiments and from the MN assays are presented as means ± SD. The results of CBMN and SCGE assays (under standard conditions and after treatment with lesion-specific enzymes) were analyzed by one-way ANOVA followed by Dunnett’s multiple comparisons test. The *t* test was used for experiments with/without S9 in TR146 cells to calculate the statistical differences between the groups after the treatment of the cells with both compounds. Differences were considered as significant when the *p* values were ≤ 0.05.

All statistical calculations are based on comparisons between results which were obtained with cells which had been treated with the test compounds and results which were obtained with corresponding solvent controls.

## Results

### Cytotoxic effects of test compounds

Since cytotoxic effects may lead to false positive results in SCGE assays (Henderson et al. 1998), several experimental series were conducted with HepG2 and TR146 cells, in which the indicator cells were exposed to different concentrations of CBD and CBDV. The results of these experiments are summarized in Figures S1 and S2 (supplementary information). It can be seen that the viability of the HepG2 was not affected when the cells were exposed to concentrations ≤ 54 µM for 3 h; the highest dose (162 µM) caused a clear effect, and the viability of the cells decreased by approximately 50%. When the treatment time was extended to 24 h, a decline of viable cells was also seen with 54 µM (Fig. S1A–D). The impact of the compounds on the viability of TR146 cells is shown in Figures S2A-B.

The vitality of the HepG2 cells in SCGE experiments was also determined with the trypan blue exclusion technique after treatment with 54 µM of CBD and CBDV (the highest dose tested in SCGE experiments) and was 90% ± 8 and 95% ± 4, respectively. The corresponding values for TR-146 cells are 91% ± 5 and 87% ± 4 (numbers indicate values obtained with three cultures ± standard deviations). Since misleading/false positives may occur in SCGE experiments only when the viability of the cells declines below 80% (Henderson et al. 1998), it can be excluded that the results which we obtained in the SCGE tests are due to acute toxic effects.

### SCGE assays with HepG2 and TR146 (standard conditions)

**Fig. 2:**
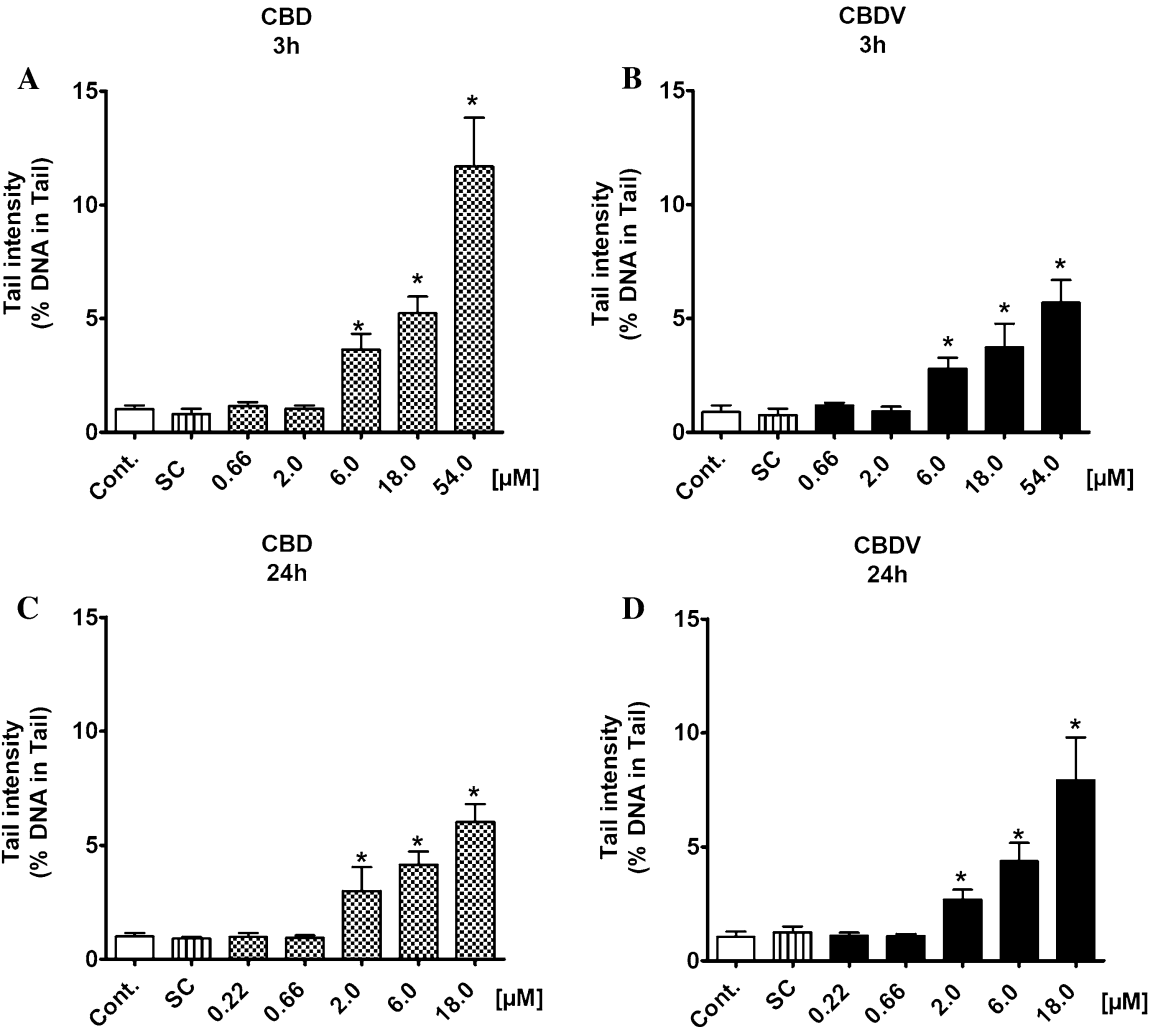
**a, b** Induction of DNA damage by CBD and CBDV in a human-derived liver cell line (HepG2). The cells were treated with different concentrations of the test compounds for 3 and 24 h. Methanol was used as a solvent control [for 3 h CBD: 1.70% (v/v) and CBDV: 1.55% (v/v); for 24 h CBD: 0.56% (v/v) for CBDV: 0.52% (v/v)]. Hydrogen peroxide (50 µM) was used as a positive control (the cells were treated for 5 min on ice) and induced clear positive effects (26.57 ± 3.64% DNA in tail). Bars indicate means ± SD of results obtained with two parallel cultures per experiment (from each culture two slides were made and 50 cells were evaluated per slide). Stars indicate statistical significance (*p* ≤ 0.05, ANOVA). All statistical calculations are based on comparisons between results which were obtained with cells which had been treated with the test compounds and results which were obtained with corresponding solvent controls

**Fig. 3:**
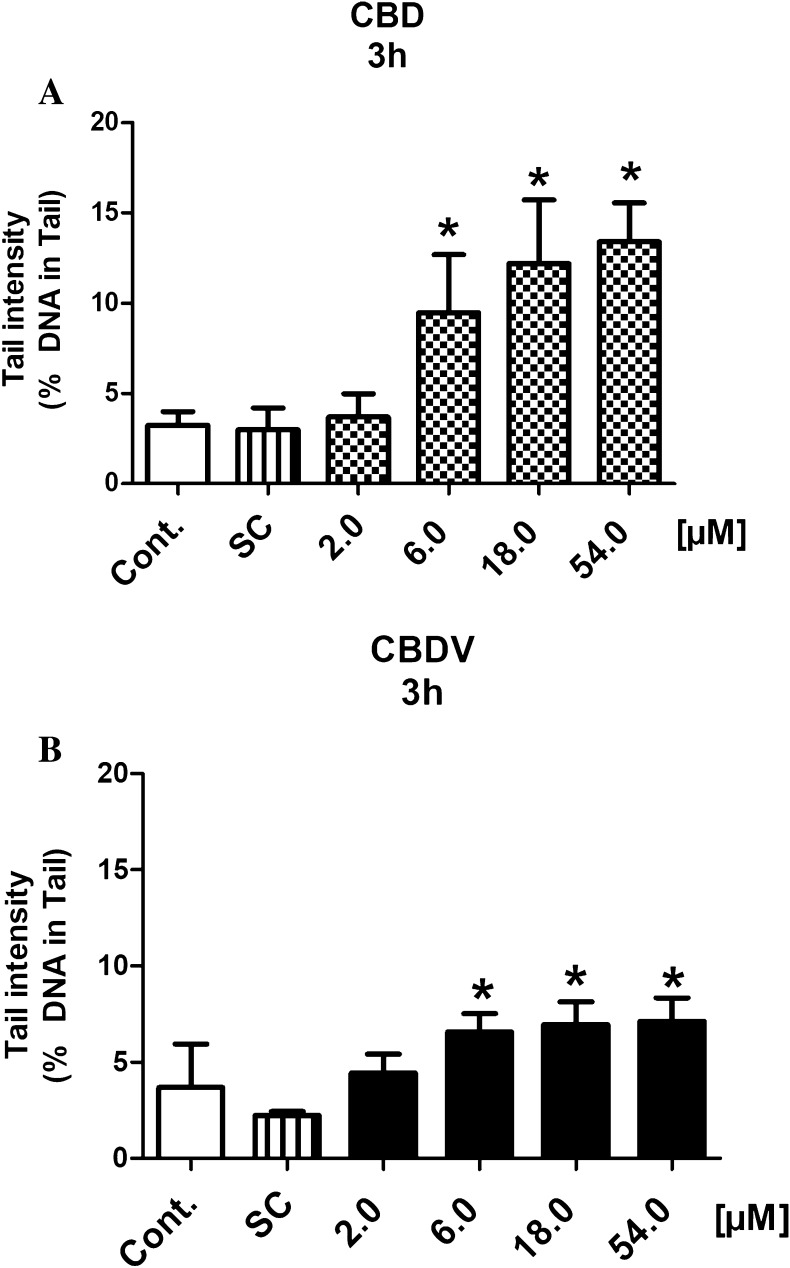
**a, b** Induction of DNA damage by CBD and CBDV in a human-derived buccal cell line (TR146). The cells were treated with different concentrations of the test compounds for 3 h. Methanol was used as solvent control [CBD: 1.70% (v/v) and CBDV: 1.55% (v/v)]. Hydrogen peroxide (50 µM) was used as a positive control (the cells were treated for 5 min on ice). The peroxide induced clear positive effects (20.12 ± 1.84% DNA in tail). Bars indicate means ± SD of results obtained with two parallel cultures per experiment (from each culture two slides were made and 50 cells were evaluated per slide). Stars indicate statistical significance (*p* ≤ 0.05, ANOVA). All statistical calculations are based on comparisons between results which were obtained with cells which had been treated with the test compounds and results which were obtained with corresponding solvent controls

**Fig. 4:**
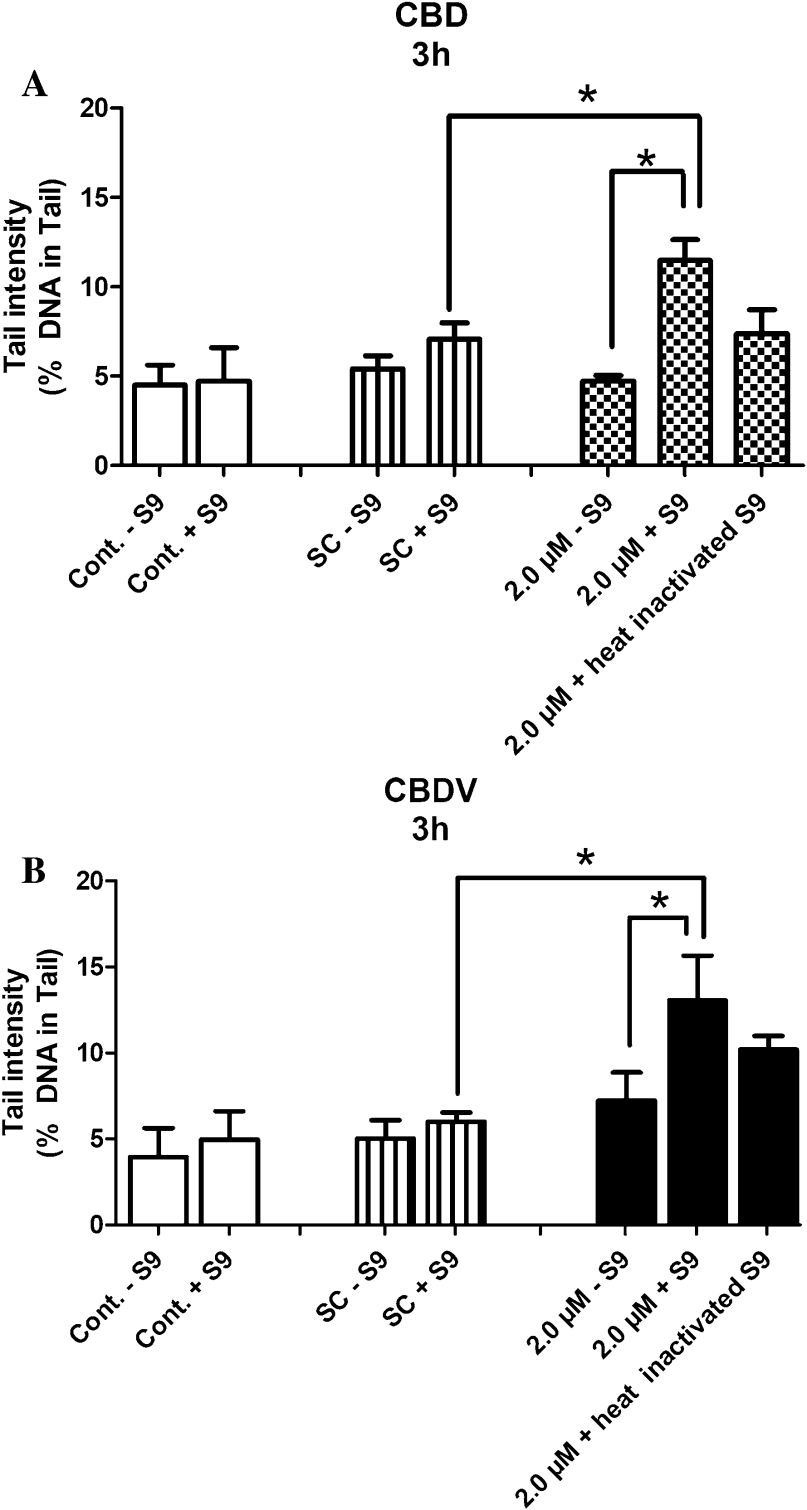
**a, b** Impact of liver enzyme homogenate on the DNA-damaging activity of CBD and CBDV in TR146 cells. The cells were treated with 2.0 µM of the cannabinoids and in parallel with liver enzyme homogenate (for details see “Materials and methods”). Bars indicate means ± SD of results obtained with two parallel cultures per experiment (from each culture two slides were made and 50 cells were evaluated per slide). Stars indicate statistical significance (*p* ≤ 0.05, Two-tailed paired *t* test). All statistical calculations are based on comparisons between results which were obtained with cells which had been treated with the test compounds and results which were obtained with corresponding solvent controls

The results of SCGE experiments with the cannabinoids are summarized in Figs. 2, 3 and 4. Results of individual experiments can be found in supplementary tables SI 1A-B. Since it is known that the genotoxic response of promutagens in HepG2 may increase after extended treatment (Natarajan and Darroudi 1991), two exposure periods (3 h and 24 h) were tested. Both drugs caused DNA damage in both cell types (HepG2 and TR146). In the liver-derived cells, significant induction of damage was seen with both compounds at concentrations ≥ 6.0 µM after 3 h (Fig. 2a, b). When the cells were treated for 24 h, clear damage was observed with the lower concentrations (≥ 2.0 µM) (Fig. 2c, d).

Also with TR146 cells, which are derived from the buccal cavity, positive findings were obtained under identical conditions, i.e., induction of comets was detected with both drugs at concentrations ≥ 6.0 µM after 3 h (Fig. 3a, b).

It is notable that CBD was more active than its propyl analogue (CBDV) in both cell lines, when the cells were exposed for 3 h, i.e., the extent of DNA damage which was seen with the former compound under identical conditions was approximately threefold higher.

To find out if the compounds are converted to mutagenic metabolites by liver enzymes, an additional experimental series was realized, in which S9 mix (which contains active phase I enzymes) was added to the incubation during the treatment of TR146 cells with the cannabinoids. The results are shown in Fig. 4a, b. Addition of the enzyme homogenate caused induction of DNA damage in TR146 cells, but no such effect was seen when the liver enzymes were inactivated by heating.

### SCGE assays with lesion-specific enzymes with HepG2

**Fig. 5:**
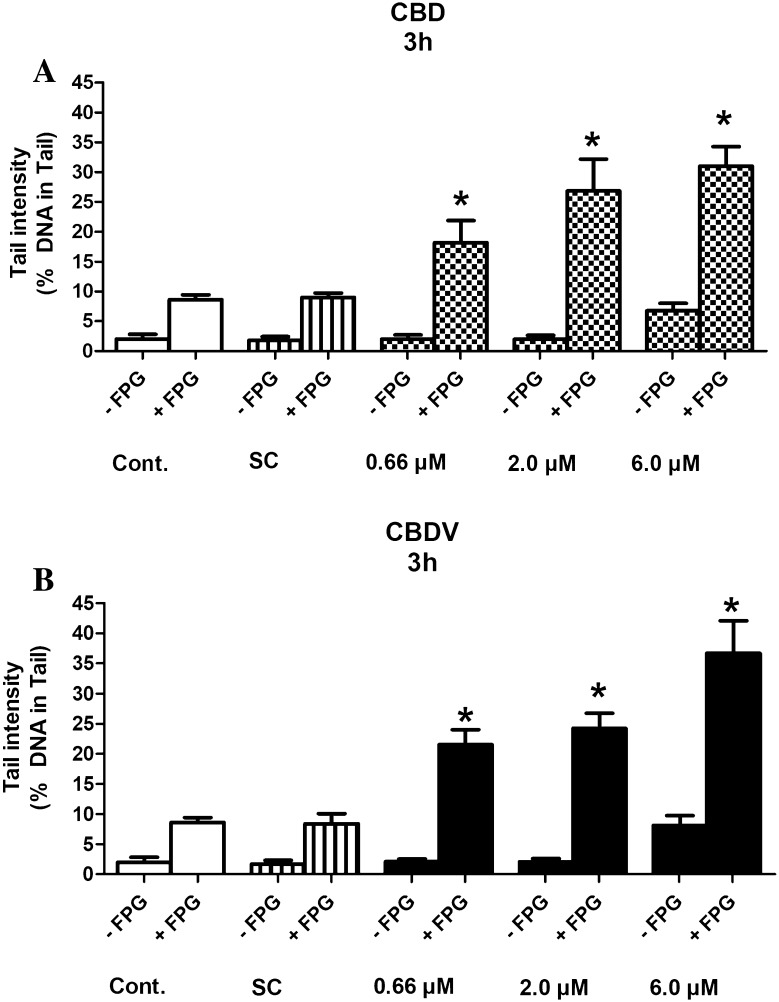
**a, b** Formation of oxidized purines in HepG2 cells by CBD and CBDV. The cells were exposed to the test compounds for 3 h. Subsequently, the nuclei were isolated after lysis and treated with FPG or with the corresponding buffers before electrophoresis for 30 min. Bars indicate means ± SD of results obtained with two cultures per experimental point. From each culture, two slides were made and 50 cells were evaluated per slide. Stars indicate statistical significance (*p* ≤ 0.05, ANOVA). All statistical calculations are based on comparisons between results which were obtained with cells which had been treated with the test compounds and results which were obtained with corresponding solvent controls

**Fig. 6:**
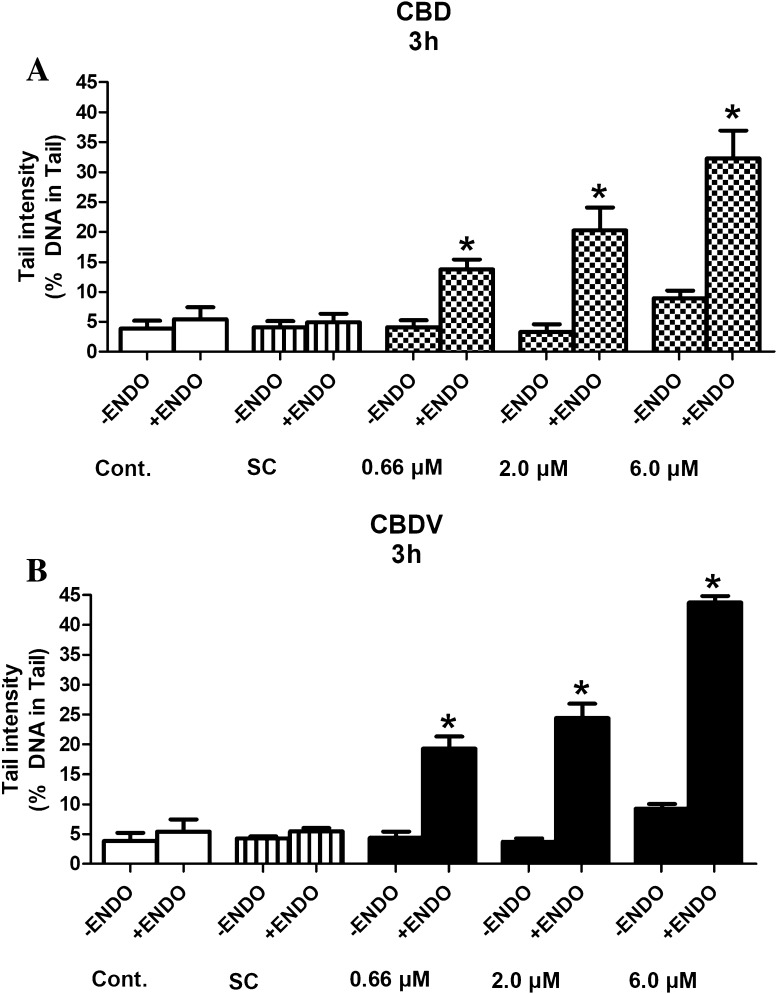
**a, b** Formation of oxidized pyrimidines in HepG2 cells by CBD and CBDV. The cells were exposed to the test compounds for 3 h. Subsequently, the nuclei were isolated after lysis and treated with ENDO III or with the corresponding buffers before electrophoresis for 45 min. Bars indicate means ± SD of results obtained with two cultures per experimental point. From each culture, two slides were made and 50 cells were evaluated per slide. Stars indicate statistical significance (*p* ≤ 0.05, ANOVA). All statistical calculations are based on comparisons between results which were obtained with cells which had been treated with the test compounds and results which were obtained with corresponding solvent controls

To elucidate if the drugs cause oxidative damage of DNA bases, experiments were conducted with lesion-specific enzymes (FPG and ENDO III). The results are summarized in Figs. 5a, b and 6a, b.

It is evident that CBD and CBDV cause oxidation of purines and pyrimidines. Even with the lowest levels (0.66 µM), significant induction of comet formation was observed.

### Cytokinesis-block micronucleus (CBMN) assays with HepG2

To find out if treatment of human liver-derived cells leads to formation of MNi, which reflect structural and numerical chromosomal aberrations, cytome MN experiments were conducted with HepG2 cells. The results are summarized in Table 1. Data from individual experiments can be found in supplementary tables SI 2A-B.

**Table 1:** Impact of the two cannabinoids on MN formation and on the rates of various nuclear aberrations in HepG2 cells

Compounds	Concentrations (µM)	CPBI	CT	BN-MN^a^	MNi^b^	Nbuds	NPBs	Necrosis	Apoptosis
		Mean ± SD	%	Mean (‰) ± SD	Mean (‰) ± SD	Mean (‰) ± SD	Mean (‰) ± SD	Mean (‰) ± SD	Mean (‰) ± SD
Neg. Ctrl	0	2.04 ± 0.03	–	5.25 ± 0.35	5.75 ± 0.35	4.75 ± 0.35	3.50 ± 0.71	6.25 ± 0.35	3.00 ± 0.71
CBD	0.07	2.00 ± 0.08	3.92	6.50 ± 1.41	6.50 ± 1.41	16.00 ± 2.12*	5.25 ± 0.35	16.25 ± 1.77*	13.50 ± 0.71*
	0.22	1.93 ± 0.04	10.60	21.00 ± 1.41*	31.00 ± 2.12*	25.50 ± 2.83*	8.50 ± 1.41*	21.00 ± 0.70*	25.25 ± 3.18*
	0.66	1.83 ± 0.04	20.22	31.25 ± 2.47*	46.25 ± 3.89*	37.25 ± 1.06*	10.00 ± 1.41*	30.75 ± 1.77*	29.00 ± 1.41*
	2.00	1.72 ± 0.01	30.76	39.25 ± 3.89*	53.25 ± 2.47*	43.00 ± 2.83*	14.00 ± 0.71*	33.50 ± 2.12*	37.25 ± 1.77*
SC^c^		1.80 ± 0.00	23.05	5.00 ± 1.41	6.25 ± 0.35	5.50 ± 1.41	3.25 ± 1.06	6.75 ± 1.06	3.00 ± 0.71
*CBDV*	0.07	1.95 ± 0.05	9.17	6.00 ± 0.71	6.00 ± 0.71	15.25 ± 1.77*	6.00 ± 2.12	15.25 ± 2.47*	13.75 ± 1.77*
	0.22	1.93 ± 0.04	10.60	26.00 ± 2.83*	29.75 ± 1.77*	36.25 ± 3.18*	10.00 ± 0.71*	18.50 ± 1.41*	21.75 ± 1.06*
	0.66	1.79 ± 0.01	24.03	32.00 ± 0.71*	45.50 ± 1.41*	40.00 ± 2.12*	13.25 ± 1.77*	24.5 ± 1.41*	28.75 ± 3.89*
	2.00	1.77 ± 0.03	25.97	41.25 ± 2.47*	51.25 ± 3.89*	45.75 ± 2.47*	16.00 ± 2.12*	34.75 ± 2.47*	30.00 ± 2.83*
SC^c^		1.81 ± 0.02	22.54	5.00 ± 00	5.75 ± 0.35	5.00 ± 0.71	3.25 ± 0.35	6.25 ± 1.06	3.00 ± 0.71
Pos. Ctrl	500 µg/mL	1.80 ± 0.01	23.54	42.25 ± 5.30*	56.75 ± 1.06*	35.50 ± 1.41*	11.75 ± 1.06*	16.25 ± 1.77*	9.25 ± 3.18

*CBPI* cytokinesis-block proliferation indices, *CT* cytostasis (%), HepG2 cells were treated with different concentrations of the test compounds for 3 h. Numbers represent results (means ± SD) obtained in two independent experiments, and in each experiment, two cultures were made per experimental point. Four slides were prepared and 2000 cells were evaluated. All statistical calculations are based on comparisons between results which were obtained with cells which had been treated with the test compounds and results which were obtained with corresponding solvent controls.

*BN–MNi* binucleated cells with micronuclei, *MNi* micronuclei, *Nbuds* nuclear buds, *NPBs* nucleoplasmatic bridges, *Neg. Ctrl* cells cultivated in medium, *SC* solvent control, *Pos. Ctrl* cyclophosphamide (500 µg/ml)

*Significant differences from solvent control values (Dunnett test, *p* ≤ 0.05)

^a^Number of binucleated cells with MN

^b^Total number of MN from binucleated cells

^c^Methanol was used as solvent control [0.06% (v/v) in experiments with CBD and 0.05% (v/v) in experiments with CBDV]

Both compounds caused induction of MNi at low concentrations (≥ 0.22 µM). Additionally, a significant increase of other nuclear anomalies (Nbuds and NPBs), as well as induction of cell death (necrosis and apoptosis) was observed after treatment with both drugs.

## Discussion

The results of the present study show that CBD and CBDV cause formation of comets (which reflect single and double strand breaks and apurinic sites), oxidation of DNA bases and induction of MN (which are formed as a consequence of structural and numerical chromosomal aberrations).

The effects were seen at concentrations which are in the range of the levels also found in the blood of users. The highest concentrations of CBD detected after smoking were between 0.25 and 2.18 µM in plasma (Haney et al. 2016; Ohlsson et al. 1986). Cells in the oral cavity of users who consume oils, sprays or smoke dried plant material may be exposed to much higher doses, but no experimental data are currently available according to our knowledge. For CBDV, exposure data from humans are missing. As shown in Table 1, we found significant induction of MN with both compounds after treatment of the cells with concentrations ≥ 0.22 µM in the present study. Furthermore, increased rates of NBuds and NPBs, which are formed as a consequence of gene amplification and dicentric chromosomes (Fenech 2007), were also detected under identical conditions.

As described in the introduction, results of older studies are available (when no CBD-containing preparations were sold on the market). They show that CBD causes induction of MN and CA in bone marrow of mice (Zimmerman and Raj 1980), while no positive results were obtained in unscheduled DNA synthesis (UDS) experiments with fibroblasts in vitro (Zimmerman et al. 1978). MN induction was found in three independent experimental series after i.p. administration of CBD; the test was in partial agreement with the U.S. EPA guidelines (Mavournin et al. 1990; OECD 2016), i.e., several doses were tested, five animals were used per group, a sufficient number of cells was evaluated and positive/negative controls were included. However, the impact of the drug on erythropoiesis, which may lead to false results and OECD #474 (Tweats et al. 2007) was not taken into account. The evidence for induction of MN is supported by results of chromosomal analyses of metaphase cells from the bone marrow which showed that i.p. administration of 10 mg/kg caused a sevenfold increase over the background (Zimmerman and Raj 1980).

The only SCGE result with CBD was published by Aviello et al. (2012) who conducted a single dose experiment with colon-derived (CaCo2) cells. The authors found no induction of DNA damage when the cells were treated with 10 µM CBD for 24 h. We did not find any results of mutagenicity studies with CBDV in the literature, while  several investigations were conducted with THC which is structurally related to both compounds (Fig. 1). Consistently negative results were obtained in microbial experiments and in in vitro studies with mammalian cells and human leukocytes (Neu et al. 1970; Stenchever and Allen 1972; Stoeckel et al. 1975; Zimmerman et al. 1978), while studies done with laboratory rodents yielded controversial findings (Stoeckel et al. 1975; Van Went 1978). In a human study, clear induction of chromosomal aberrations was found in lymphocytes of individuals who consumed the alkaloid orally (Nichols et al. 1974).

The results of experiments with lesion-specific enzymes (Figs. 5a, b, 6a, b) show that both compounds cause oxidative damage of purines and pyrimidines. In this context, it is notable that pro- as well as antioxidant effects of CBD have been described. For example, the neuroprotective effects of CBD towards alcohol-induced toxicity were attributed to its antioxidant properties (Hamelink et al. 2005). Protective effects seen in LPS-stimulated macrophages were explained by inhibition of formation of pro-inflammatory cytokines, which cause formation of free oxygen radicals (Rajan et al. 2016). A molecular explanation for the antioxidant properties of CBD can be found in a publication of Borges et al. (2013). On the other hand, it was shown that CBD induces oxidative stress via activation of caspase-8 leading to apoptosis (Wu et al. 2008). Furthermore, induction of cyclooxygenase 2 (COX-2) was found in Zucker diabetic fatty rats, which leads to formation of pro-inflammatory prostaglandins and reactive oxygen species (ROS) (Wheal et al. 2014).

Our results with liver enzyme homogenate (Fig. 4) suggest that drug-metabolizing enzymes (in particular CYPs which are contained in the enzyme mix) increase the genotoxic properties of CBD and CBDV. It is well-documented, that different CYPs (in particular CYP1A1, 1A2 and 3A4) catalyze the formation of hydroxyl derivatives of CBD (Ujvary and Hanus 2016), but the mutagenic properties of these metabolites have not been investigated so far.

The most relevant result of the present investigation is the detection of MN induction by CBD and CBDV at low, physiologically relevant concentrations. MNi are formed as a consequence of chromosomal damage and it is well-documented, that increased rates in lymphocytes of humans are indicative for cancer risks (Bonassi et al. 2007). The results of the present experiments and also the findings of Zimmerman and Raj (1980), who found induction of MN and CA in vivo in bone marrow of mice, indicate that CBD is a potent mutagen. The International Committee on Harmonized Guidance on Genotoxicity Testing of Pharmaceuticals states in a position paper very clearly that “unequivocally genotoxic compounds in the absence of other data are presumed to be trans-species carcinogens, implying a hazard in humans. Such compounds need to be subjected to long-term carcinogenicity studies” (Muller et al. 1999). Furthermore, it should be also explored if sperm abnormalities, which may be also caused by genomic instability and were induced by CBD in mice (Zimmerman and Zimmerman 1990), are due to DNA damage and may lead to infertility of users. As mentioned above, no data from long-term carcinogenicity experiments with rodents are available at present. It is notable in this context that it was found that the sensitivity of a combination of positive MN assays with rodents and in vitro SCGE assays for the detection of group 1 carcinogens (IARC) was found to be 95.6% (Bhagat 2018). In regard to the MN data obtained in bone marrow cells, it will be relevant to investigate if the drugs induce alterations of the erythropoetic system (see above) and also if inhalative and oral exposure cause adverse effects. Additional experiments to elucidate the molecular mechanisms by which the cannabinoids cause damage of the genetic material would also contribute to a better understanding of their possible health risks in humans.

## Electronic supplementary material

Below is the link to the electronic supplementary material.

Supplementary material 1 (DOCX 194 KB)

## Abbreviations

BN-MNi: Binucleated cells with micronuclei
CA: Chromosomal aberration
CBD: Cannabidiol
CBDV: Cannabidivarin
CBMN assay: Cytokinesis-block micronucleus assay
CBPI: Cytokinesis-block proliferation index
CT: Cytostasis
CP: Cyclophosphamide
MN: Micronucleus
MNi: Micronuclei
Nbuds: Nuclear buds
NPBs: Nucleoplasmatic bridges
ROS: Reactive oxygen species
SCGE: Single cell gel electrophoresis
