# Supplementary material for: Low doses of widely consumed cannabinoids (cannabidiol and cannabidivarin) cause DNA damage and chromosomal aberrations in human-derived cells
Source: Arch Toxicol. 2018 Oct 19;93(1):179–88. doi: 10.1007/s00204-018-2322-9 (PMC6342871; doi:10.1007/s00204-018-2322-9)
Supplement: Supplementary file 1 — Supplementary material 1 (DOCX 194 KB) [file 204_2018_2322_MOESM1_ESM.docx]

**Supporting Information**

**Low doses of widely consumed cannabinoids (cannabidiol and cannabidivarin) cause DNA damage and chromosomal aberrations in human derived cells**

Chiara Russo^1^, Franziska Ferk^2^, Miroslav Mišík^2^, Nathalie Ropek^2^, Armen Nersesyan^2^, Doris Mejri^2^, Klaus Holzmann^2^, Margherita Lavorgna^1^, Marina Isidori^1^, Siegfried Knasmüller^2,a^

^1^Dipartimento di Scienze e Tecnologie Ambientali, Biologiche e Farmaceutiche, Università della Campania, L. Vanvitelli, Via Vivaldi 43, I-81100 Caserta, Italy

^2^Institute of Cancer Research, Department of Internal Medicine 1, Medical University of Vienna, 1090 Vienna, Austria

^a^Corresponding author: Institute of Cancer Research, Department of Internal Medicine 1, Medical University of Vienna, Borschkegasse 8A, 1090 Vienna, Austria.

Tel: +43-1-40160-57562; Fax: +43-1-40160-957500.

E-mail: siegfried. knasmueller@meduniwien.ac.at

**Figure S1A-D**

**Fig. S1.** Impact of treatment with CBD and CBDV on the viability of HepG2 cells. The cells were exposed to methanolic solutions of the drugs for different time periods. Methanol was tested as solvent control (SC, CBD: 1.70% and CBDV: 1.55%). Subsequently, their viability was determined with a CASY^®^ cell counter. Bars indicate means ± SD of three independent experiments (one culture per experiment). Stars indicate statistical significance (*p* ≤ 0.05, ANOVA).

**Figure S2A-B**

**Fig. S2.** Impact of CBD and CBDV on the viability of TR-146 cells. The cells were exposed (3h) to methanolic solutions of the drugs. Methanol was tested as solvent control (SC, CBD: 1.70% and CBDV: 1.55%). Subsequently, their viability was determined with a CASY^®^ cell counter. Bars indicate means ± SD of three independent experiments (one culture per experiment).

**Table S1A.** Statistical analyses of SCGE data from two independent experiments with CBD in HepG2 cells.

| **Assay**  **(incubation time)** | **Cont.** | **SC (Methanol)** | **0.22 µM** | **0.66 µM** | **2.0 µM** | **6.0 µM** | **18.0 µM** | **54.0 µM** |
| --- | --- | --- | --- | --- | --- | --- | --- | --- |
| ***SCGE***  ***(3h)*** |  |  |  |  |  |  |  |  |
| **Experiment 1**  Mean  CI  Ranges | 0.91  0.65-1.16  0.79-1.13 | 0.80  0.42-1.17  0.45-0.95 | n. t. | 1.15  0.86-1.44  0.93-1.32 | 1.08  0.84-1.33  0.94-1.25 | 3.20  2.51-3.90  2.78-3.71 | 4.65  4.37-4.93  4.48-4.86 | 10.83  11.15-10.52  10.60-11.06 |
| **Experiment 2**  Mean  CI  Ranges | 1.11  0.85-1.36  0.95-1.32 | 0.85  0.59-1.10  0.74-1.08 | n. t. | 1.05  0.99-1.11  1.00-1.10 | 1.01  0.79-1.23  0.92-1.21 | 4.05  2.94-5.16  3.03-4.60 | 5.81  4.98-6.65  5.20-6.26 | 12.54  7.86-17.23  9.43-15.84 |
| ***SCGE***  ***(24h)*** |  |  |  |  |  |  |  |  |
| **Experiment 1**  Mean  CI  Ranges | 1.03  0.96-1.10  0.97-1.07 | 0.90  0.79-1.02  0.84-1.01 | 0.97  0.67-1.27  0.79-1.14 | 0.67  0.13-1.47  0.09-1.14 | 3.96  3.64-4.27  3.70-4.17 | 4.47  3.59-5.37  4.03-5.22 | 5.47  4.30-6.65  4.85-6.50 | n. t. |
| **Experiment 2**  Mean  CI  Ranges | 0.96  0.59-1.32  0.76-1.25 | 0.94  0.52-1.36  0.67-1.29 | 0.96  0.68-1.26  0.80-1.23 | 0.94  0.77-1.11  0.83-1.07 | 2.01  1.88-2.15  1.92-2.11 | 3.80  3.13-4.48  3.23-4.14 | 6.57  6.21-6.93  6.39-6.90 | n. t. |

CI, confidence interval; n. t. , not tested; SC, solvent control; SCGE single cell gel electrophoresis assay.

**Table S1B.** SCGE data from two independent experiments with CBDV in HepG2 cells.

| **Assay**  **(incubation time)** | **Cont.** | **SC (Methanol)** | **0.22 µM** | **0.66 µM** | **2.0 µM** | **6.0 µM** | **18.0 µM** | **54.0 µM** |
| --- | --- | --- | --- | --- | --- | --- | --- | --- |
| ***SCGE***  ***(3h)*** |  |  |  |  |  |  |  |  |
| **Experiment 1**  Mean  CI  Ranges | 0.67  0.33-1.01  0.52-0.98 | 0.75  0.30-1.21  0.56-1.18 | n. t. | 1.18  0.99-1.37  1.01-128 | 1.05  0.91-1.20  0.93-1.15 | 3.01  2.25  3.77 | 4.55  3.39-5.71  3.61-5.38 | 5.50  3.36-7.63  4.07-6.78 |
| **Experiment 2**  Mean  CI  Ranges | 1.11  0.85-1.36  0.95-1.32 | 0.87  0.52-1.21  0.64-1.13 | n. t. | 1.23  0.78-1.67  1.03-1.64 | 0.77  0.43-1.12  0.51-0.98 | 2.57  1.89-3.26  1.97-2.99 | 2.90  2.21-3.59  2.46-3.41 | 5.89  4.91-6.88  5.40-6.8 |
| ***SCGE***  ***(24h)*** |  |  |  |  |  |  |  |  |
| **Experiment 1**  Mean  CI  Ranges | 1.15  0.87-1.43  0.94-1.36 | 1.24  0.83-1.66  0.87-1.44 | 1.10  0.91-1.30  0.96-1.25 | 1.11  0.92-1.29  0.97-1.24 | 2.47  1.98-2.93  2.09-2.82 | 4.51  3.36-5.68  3.89-5.40 | 9.36  7.90-10.82  8.52-10.57 | n. t. |
| **Experiment 2**  Mean  CI  Ranges | 0.96  0.60-1.13  0.76-1.25 | 1.22  0.77-1.68  0.97-1.61 | 0.96  0.80-1.12  0.82-1.05 | 1.07  0.92-1.23  1.00-1.21 | 2.91  2.18-3.65  2.62-3.60 | 4.22  2.72-5.72  3.45-5.55 | 6.50  4.30-8.70  5.15-8.41 | n. t. |

CI, confidence interval; n. t. , not tested; SC, solvent control; SCGE single cell gel electrophoresis assay.

**Table S2A.** MN assay data from 2 independent experiments with CBD in HepG2 cells. ^a^

| **Drug (incubation time)** | **Pos. Cont.**  **(CP)** | **Neg. Cont.** | **SC (Methanol)** | **0.07 µM** | **0.22 µM** | **0.66 µM** | **2.0 µM** |
| --- | --- | --- | --- | --- | --- | --- | --- |
| ***CBD (3h)*** |  |  |  |  |  |  |  |
| ***Experiment 1***  *CPBI* **^b^**  **BN-MN** | *1.79*  38.5 | *2.02*  5.5 | *1.80*  6.0 | *1.94*  7.5 | *1.90*  22.0 | *1.80*  29.5 | *1.71*  36.5 |
| **MNi**  **Nbuds**  **NPBs**  **Necrosis**  **Apoptosis**  ***Experiment 2***  *CPBI* **^b^**  **BN-MN**  **MNi**  **Nbuds**  **NPBs**  **Necrosis**  **Apoptosis** | 56.0  36.5  11.0  15.0  7.0  *1.80*  46.0  57.5  34.5  12.5  17.5  11.5 | 5.5  4.5  3.0  6.0  3.5  *2.06*  5.0  6.0  5.0  4.0  6.5  2.5 | 6.5  6.5  2.5  7.5  2.5  *1.80*  4.0  6.0  4.5  4.0  6.0  3.5 | 7.5  14.5  5.0  15.0  13.0  *2.06*  5.5  5.5  17.5  5.5  17.5  14.0 | 32.5  27.5  9.5  20.5  23.0  *1.96*  20.0  29.5  23.5  7.5  21.5  27.5 | 49.0  38.0  11.0  29.5  28.0  *1.86*  33.0  43.5  36.5  9.0  32.0  30.0 | 55.0  45.0  14.5  32.0  38.5  *1.73*  42.0  51.5  41.0  13.5  35.0  36.0 |

^a^ mean values per 1000 cells per experimental point.

^b^ proliferation indices were calculated from 500 cells.

**Table S2B.** MN assay data from 2 independent experiments with CBDV in HepG2 cells. ^a^

| **Drug (incubation time)** | **Pos. Cont.**  **(CP)** | **Neg. Cont.** | **SC (Methanol)** | **0.07 µM** | **0.22 µM** | **0.66 µM** | **2.0 µM** |
| --- | --- | --- | --- | --- | --- | --- | --- |
| ***CBDV (3h)*** |  |  |  |  |  |  |  |
| ***Experiment 1***  *CPBI* **^b^**  **BN-MN** | *1.79*  38.5 | *2.02*  5.5 | *1.82*  5.0 | *1.91*  6.5 | *1.90*  24.0 | *1.78*  31.5 | *1.75*  39.5 |
| **MNi**  **Nbuds**  **NPBs**  **Necrosis**  **Apoptosis**  ***Experiment 2***  *CPBI* **^b^**  **BN-MN**  **MNi**  **Nbuds**  **NPBs**  **Necrosis**  **Apoptosis** | 56.0  36.5  11.0  15.0  7.0  *1.80*  46.0  57.5  34.5  12.5  17.5  11.5 | 5.5  4.5  3.0  6.0  3.5  *2.06*  5.0  6.0  5.0  4.0  6.5  2.5 | 6.0  5.5  3.5  5.5  2.5  *1.79*  5.0  5.5  4.5  3.0  7.0  3.5 | 6.5  14.0  7.5  13.5  15.0  *1.98*  5.5  5.5  16.5  4.5  17.0  12.5 | 28.5  38.5  9.5  17.5  21.0  *1.96*  28.0  31.0  34.0  10.5  19.5  22.5 | 46.5  41.5  12.0  25.5  26.0  *1.80*  32.5  44.5  38.5  14.5  23.5  31.5 | 54.0  47.5  14.5  33.0  28.0  *1.79*  43.0  48.5  44.0  17.5  36.5  32.0 |

^a, b,^  described in Table S2A.
